# Quantitative evaluation of diffusion-weighted MRI for differentiating benign and malignant thyroid nodules larger than 4 cm

**DOI:** 10.1186/s12880-023-01141-z

**Published:** 2023-12-13

**Authors:** Tingting Zheng, Xiaoli Xie, Zhaoxian Ni, Lang Tang, Pu-Yeh Wu, Bin Song

**Affiliations:** 1 Department of Radiology, Minhang Hospital, Fudan University, No 170, Xinsong Road, Minhang District, Shanghai, 201199 China; 2 Department of Pathology, Minhang Hospital, Fudan University, No 170, Xinsong Road, Minhang District, Shanghai, 201199 China; 3 Department of General Surgery, Minhang Hospital, Fudan University, No 170, Xinsong Road, Minhang District, Shanghai, 201199 China; 4 Department of Ultrasound, Minhang Hospital, Fudan University, No 170, Xinsong Road, Minhang District, Shanghai, 201199 China; 5 GE Healthcare, MR Research China, Beijing, China

**Keywords:** Diffusion-weighted imaging, Quantitative evaluation, Large thyroid nodule, Benign and malignant

## Abstract

**Purpose:**

Our study aimed to diagnose benign or malignant thyroid nodules larger than 4 cm using quantitative diffusion-weighted imaging (DWI) analysis.

**Methods:**

Eighty-two thyroid nodules were investigated retrospectively and divided them into benign (*n* = 62) and malignant groups (*n* = 20). We calculated quantitative features DWI and apparent diffusion coefficient (ADC) signal intensity standard deviation (DWI_SD_ and ADC_SD_), DWI and ADC signal intensity ratio (DWI_SIR_ and ADC_SIR_), mean ADC and minimum ADC value (ADC_mean_ and ADC_min_) and ADC value standard deviation (ADC_VSD_). Univariate and multivariate logistic regression were conducted to identify independent predictors, and develop a prediction model. We performed receiver operating characteristic (ROC) analysis to determine the optimal threshold of risk factors, and constructed combined threshold models. Our study calculated diagnostic performance including area under the ROC curve (AUC), accuracy, sensitivity, specificity, positive predictive value (PPV), negative predictive value (NPV), and unnecessary biopsy rate of all models were calculated and compared them with the American College of Radiology Thyroid Imaging Reporting and Data System (ACR-TIRADS) result.

**Results:**

Two independent predictors of malignant nodules were identified by multivariate analysis: DWI_SIR_ (*P* = 0.007) and ADC_min_ (*P* < 0.001). The AUCs for multivariate prediction model, combined DWI_SIR_ and ADC_min_ thresholds model, combined DWI_SIR_ and ADC_SIR_ thresholds model and ACR-TIRADS were 0.946 (0.896–0.996), 0.875 (0.759–0.991), 0.777 (0.648–0.907) and 0.722 (0.588–0.857). The combined DWI_SIR_ and ADC_min_ threshold model had the lowest unnecessary biopsy rate of 0%, compared with 56.3% for ACR-TIRADS.

**Conclusion:**

Quantitative DWI demonstrated favorable malignant thyroid nodule diagnostic efficacy. The combined DWI_SIR_ and ADC_min_ thresholds model significantly reduced the unnecessary biopsy rate.

**Supplementary Information:**

The online version contains supplementary material available at 10.1186/s12880-023-01141-z.

## Main points

- The multivariate prediction model demonstrated satisfactory diagnostic performance with an AUC of 0.946 (0.896–0.996).
- Combined DWI_SIR_ and ADC_min_ thresholds model demonstrated a high specificity with an unnecessary biopsy rate of 0%.
- The multivariate prediction model and the combined threshold models are better than the ACR-TIRADS.

## Introduction

Thyroid nodules manifest in up to 50–60% of the general population as detected by high-resolution ultrasound, while only around 10% of these nodules are malignant [1–3]. Since 2014, the overall incidence of thyroid cancer incidence rate has decreased, but the incidence and mortality rate of tumors larger than 4 cm are still rising [4]. Thyroid nodules larger than 4 cm are important for surgical decision-making in adult patients with thyroid nodules, according to the 2017 Thyroid Cancer Staging Manual of the American Joint Committee on Cancer (AJCC) [5]. Ultrasonography (US) is currently the main imaging technique for evaluating thyroid nodules [6–9]. US characteristics of thyroid nodules, such as the American College of Radiology Thyroid Imaging Reporting and Data System (ACR-TIRADS), have been employed for risk stratification [10]. However, the interobserver agreement on the TI-RADS remains only fair to moderate [11, 12] and these methods mainly focus on thyroid nodules smaller than 4 cm. Although biopsy is regarded as the gold standard for the preoperative diagnosis of thyroid cancer, it has reduced sensitivity when applied to thyroid nodules larger than 4 cm [13–15].

Diffusion-weighted imaging (DWI), a non-contrast magnetic resonance imaging (MRI) technology, detects water molecule random mobility and offers information on tissue microstructure and cell density. The apparent diffusion coefficient (ADC) map can be further calculated from DWI to quantify the diffusion characteristic of tissues [16, 17]. DWI was used to diagnose benign and malignant tumors [18–21]. Although earlier research has demonstrated its effectiveness of DWI in distinguishing between benign and malignant thyroid nodules, those studies have included nodules of all sizes [22, 23]. For larger lesions, DWI has a superior diagnostic value, but there were limited researcher on the diagnostic value of DWI for thyroid nodules larger than 4 cm. Meanwhile, accurate pre-operative assessment of thyroid nodules is crucial for subsequent treatment. Accordingly, it is important to distinguish between benign and malignant nodules larger than 4 cm before surgery [24].

Consequently, this study aimed to evaluate approaches using quantitative DWI, and compare them with ACR-TIRADS to differentiate between benign and malignant thyroid nodules larger than 4 cm preoperatively.

## Materials and methods

### Patients and study design

The study followed the Declaration of Helsinki (revised 2013). The Institutional Ethics Committee of Minhang Hospital affiliated with Fudan University approved this observational, retrospective study (approval number: 2021–008-01 K) with a waiver of informed consent.

**Fig. 1:**
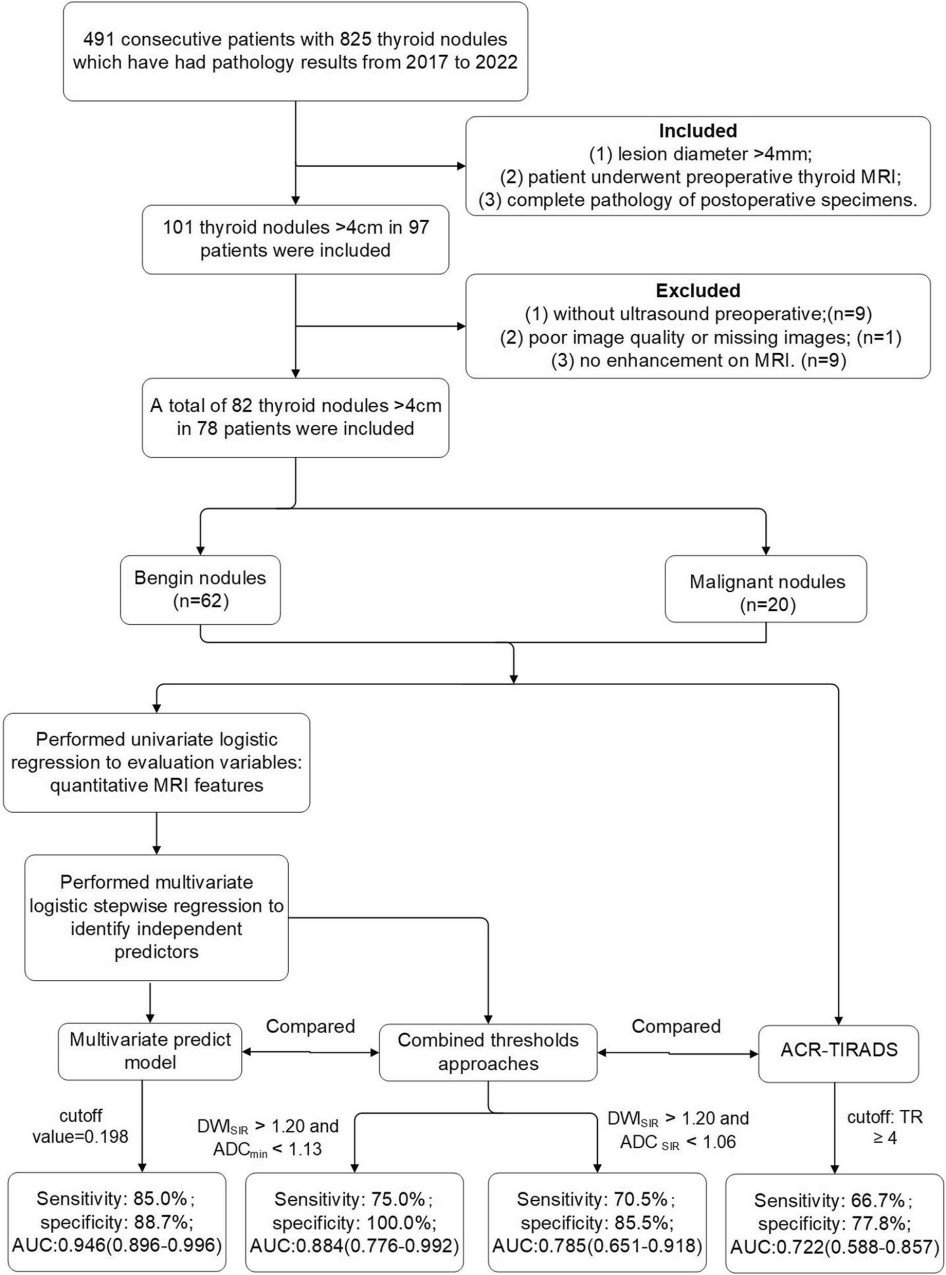
The study flowchart. Abbreviations: MRI, Magnetic resonance imaging; DWI, diffusion weighted imaging; ADC, apparent diffusion coefficient; SIR, signal intensity rate; ADC_min_, minimum apparent diffusion coefficient value; ACR-TIRADS, American College of Radiology Thyroid Imaging Reporting and Data System; TR, TI-RADS; AUC, area under the receiver operating characteristic curve

We reviewed consecutive patients with thyroid nodules who had pathology results at our institution between 2017 and 2022. The inclusion criteria included: 1) lesion diameter larger than 4 cm; 2) patients who underwent preoperative thyroid MRI; 3) complete pathology of postoperative specimens. The exclusion criteria included: 1) incomplete clinical and imaging data; 2) poor image quality; 3) lack of contrast enhancement on MRI. Figure 1 displays the study flowchart.

Finally, 82 lesions from 78 patients (32 males and 50 females; age: 50.26 ± 16.20 years; age range: 15–78 years) met the inclusion criteria. Lesions were categorized into benign (*n* = 62) and malignant (*n* = 20) groups according to the postoperative pathology.

### MRI acquisition

The 1.5 T MRI scanner (Excite HD; GE Healthcare, Waukesha, WI, USA) used for all MRI examinations was set up with an 8-channel customized neck surface coil (Chenguang Medical Technology Ltd, Shanghai, China). The scan covered the thoracic inlet to the base of the cranium were covered by the scan. The MRI sequences used (CE-T1WI) included axial and coronal fat-suppressed T2-weighted imaging (T2WI), axial T1-weighted imaging (T1WI), single-shot spin-echo echo-planar imaging (SS-SE-EPI) DWI at b values of 0 and 800 s/mm^2^, and axial multiphasic contrast-enhanced T1WI comprised the MRI sequences used (CE-T1WI). A gadolinium contrast agent (Magnevist; Bayer Healthcare, Berlin, Germany) was injected for the CE-T1WI acquisition at a dose of 0.2 mL/kg and a rate of 3 mL/s, followed immediately by 20 mL of physiological saline flushing. Following the injection of the contrast agent, six phases were recorded at intervals of 30, 60, 120, 180, 240, and 300 s intervals while the patients were asked to hold their breath. Table S1 lists detailed acquisition parameters.

### Image analysis

**Fig. 2:**
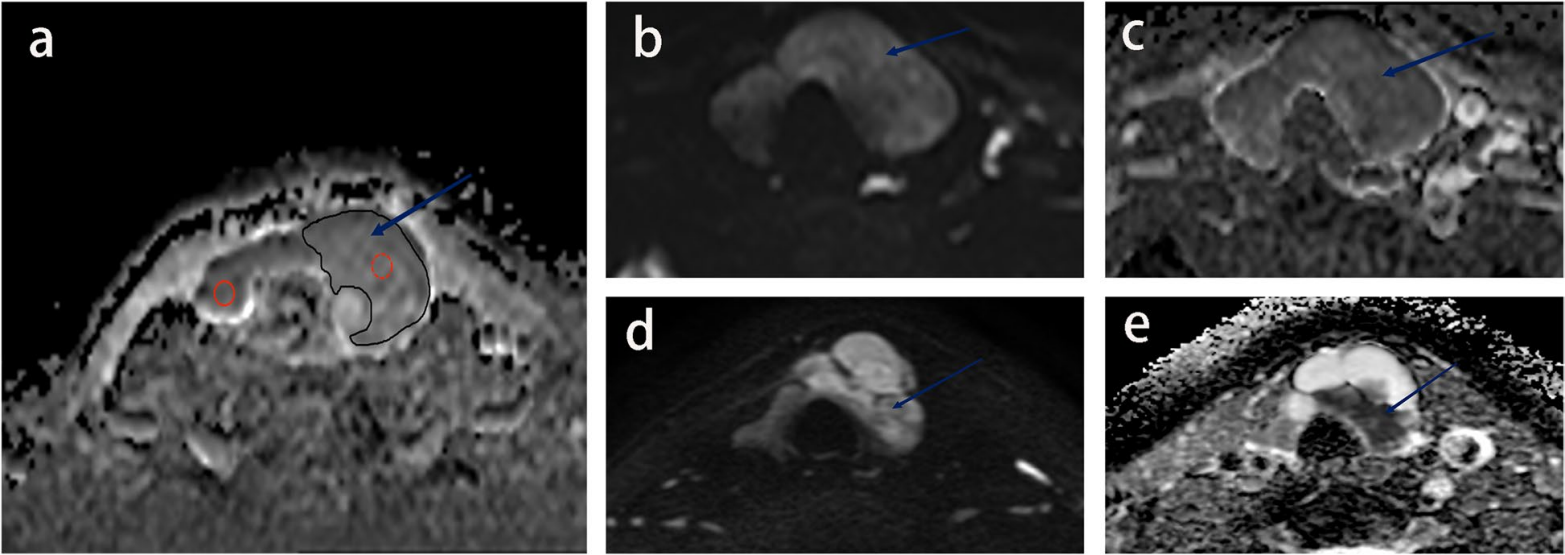
Representative DWI quantitative measurements. Lesions as indicated by blue arrows. **a** shows an example diagram of the ROI sketch. The black line outlines ROI 1, which is the whole solid portion of the slice with maximum transverse diameter for the lesion. The red line outlines ROI 2 of 8-10 mm^2^, the red dashed line is the interior of the lesion, and the red solid line is the contralateral-normal thyroid tissue. **b** and **c** show a patient with thyroid adenoma. **b** shows a DWI image with DWI_SD_ and DWI_SIR_ of 22 and 1.07. **c** shows ADC images with ADC_SD_, ADC_SIR_, ADC_min_, ADC_mean_ and ADC_VSD_ of 110, 0.97, 1.15, 152 and 171. **d** and **e** show a patient with papillary thyroid cancer. d is a DWI image with DWI_SD_ and DW_SIR_ of 24.7 and 1.56. e is an ADC image with ADC_SD_, ADC_SIR_, ADC_min_, ADC_mean_ and ADC_VSD_ of 83, 0.74, 0.65, respectively, 1.04 and 69.59, respectively. Abbreviations: DWI, diffusion weighted imaging; ADC, apparent diffusion coefficient; SI, signal intensity; SD, standard deviation; ADC_min_, minimum apparent diffusion coefficient value; ADC_mean_, mean apparent diffusion coefficient value; ADC_VSD_, standard deviation of apparent diffusion coefficient value

ADC maps were automatically created from DWI images (b = 0 and 800 s/mm^2^) on the console using mono-exponential fitting. Quantitative DWI parameters were measured by two MRI diagnosticians who were blind to the lesion pathology (a chief physician with eight years of experience and a resident with one year each in thyroid MRI diagnosis), using picture archiving and communication system (PACS) and Advantage Workstation 4.5 (GE Healthcare, Waukesha, WI, USA). The section of the whole solid leision portion of the lesion with maximum transverse diameter (excluding cystic, hemorrhage, necrosis, calcium, and vascular structures) was selected to delineate the first region of interest (ROI 1). The following quantitative features in the ROI 1 were measured: 1) mean DWI signal intensity (DWI_SI_); 2) mean ADC signal intensity (ADC_SI_); 3) mean ADC value (ADC_mean_) and minimum ADC value (ADC_min_). Another ROI with an 8–10 mm^2^ area is also outlined as a relatively homogeneous solid part without cystic, hemorrhage, necrosis, calcium and vascular structures in the lesion and contralateral to the normal thyroid tissue. The following quantitative features of the ROI 2 were measured in the lesion: 1) DWI signal intensity standard deviation (DWI_SD_) and ADC signal intensity standard deviation (ADC_SD_) and ADC value standard deviation (ADC_VSD_); 2) mean DWI signal intensity and mean ADC signal intensity of contralateral normal thyroid tissue (DWI_NSI_ and ADC_NSI_). The following formulas, DWI_SIR_ = DWI_SI_ / DWI_NSI_ and ADC_SIR_ = ADC_SI_ / ADC_NSI,_ were used to calculate the DWI signal intensity rate (DWI_SIR_) and ADC signal intensity rate (ADC_SIR_). DWI images (b = 800 s/mm^2^) and ADC map generated from DWI images (b = 0 and 800 s/mm^2^) were used for quantitative parameters extraction. All measurements were performed twice and averaged. Figure 2a demonstrates representative images of ROI delineation.

### ACR-TIRADS

Two US experts retrospectively reviewed US images of thyroid lesions, reaching a consensus without knowledge of the lesion pathology. All lesions with category ≥ 4 were considered malignant according to ACR-TIRADS.

### Statistical analysis

SPSS statistical software (version 26.0; IBM Corp, Armonk, NY, USA) and MedCalc (version 20.100; MedCalc Software, Ostend, Belgium) were used for all statistical analyses, and *P* values < 0.05 were deemed statistically significant. Quantitative MRI parameters and categorical variables of the malignant and benign groups were compared using independent t-tests, Chi-square tests, and Fisher's exact tests, respectively. Interobserver agreement was assessed using the intraclass correlation coefficient (ICC).

The malignancy prediction model was built using independent factors that were found using univariate and multivariate logistic stepwise regression. By optimizing the Youden's index, receiver operating characteristic (ROC) curve analysis was used to determine the ideal threshold values for the pertinent parameters. Combined thresholds approaches were established based on malignancy-related parameters (Supplementary Method). Individual parameters and models were evaluated using ROC curves, with the area under the ROC curve (AUC) compared by the DeLong test. Unnecessary biopsy rate was defined as the percentage of benign lesions for those requiring biopsy. The diagnostic performance measures for each model, including as accuracy, sensitivity, specificity, positive predictive value (PPV), negative predictive value (NPV), and unnecessary biopsy rate were calculated, comparing the ACR-TIRADS result was compared.

## Results

### Clinicopathological characteristics

Table 1 lists the clinicopathological features of thyroid nodules. Except for the location (*P* = 0.015), there was no difference in the distribution of other features in the benign and malignant thyroid nodules. Table S2 demonstrates the pathological types of thyroid nodules.

**Table 1:** Clinicopathologic characteristics

Characteristic	Benign	Malignant	Total	*P* value
Age (years)	50.7 ± 15.1	48.8 ± 19.6	50.3 ± 16.2	0.636
Gender				0.092
Male	21 (33.9)	11 (55.0)	32 (39.0)	
Female	41 (66.1)	9 (45.0)	50 (61.0)	
Lesion number				0.306
Unifocal	23 (37.1)	10 (50.0)	33 (40.2)	
Multifocal	39 (62.9)	10 (50.0)	49 (49.8)	
Location				0.015*
Left lobe	35 (56.5)	4 (20.0)	39 (47.6)	
Right lobe	25 (40.3)	14 (70.0)	39 (47.6)	
Isthmus	2 (2.4)	2 (10.0)	4 (4.9)	

The data are presented as number of patients with the percentage in parentheses

*Abbreviations*: *SD* Standard deviation

* *P* < 0.05

### Diagnostic performance of quantitative parameters

**Fig. 3:**
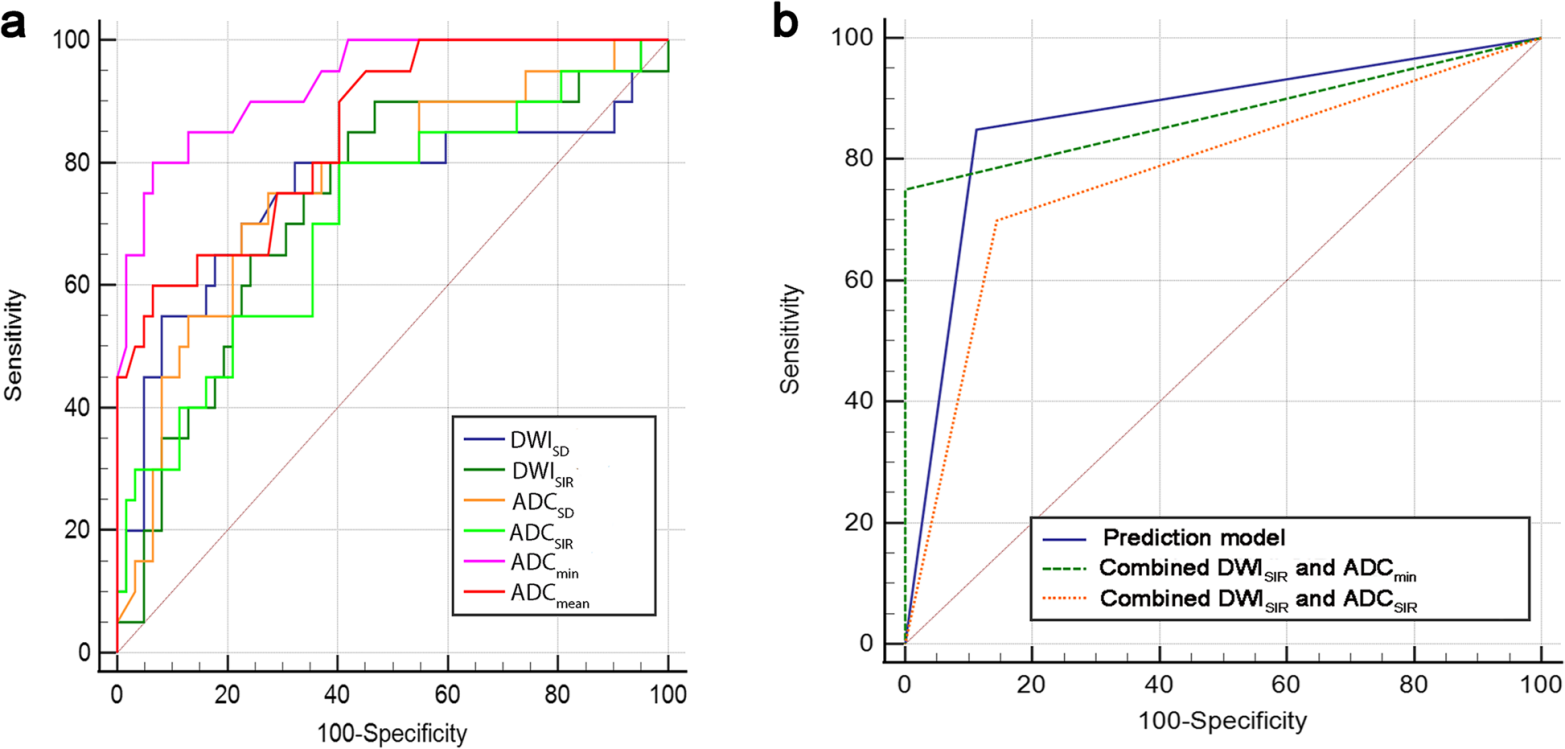
The ROC curves. **a** is ROC curves of meaningful single parameters. **b** is multivariate prediction model, combined thresholds models and ACR-TIRADS for malignant and benign thyroid nodules. Abbreviations: DWI, diffusion weighted imaging; ADC, apparent diffusion coefficient; SD, standard deviation; SIR, signal intensity rate; ADCmin, minimum value of apparent diffusion coefficient; ADCmean, mean apparent diffusion coefficient value; ADCVSD, standard deviation of apparent diffusion coefficient value

Figure 2 shows representative DWI images and the ROI delineation. Moreover, Table 2 demonstrates the results of the univariate and multivariate logistic regression analyses in predicting malignant thyroid nodules. Malignant nodules displayed significantly greater DWI_SD_ (*P* = 0.002) and DWI_SIR_ (*P* = 0.007) than benign nodules. Additionally, malignant nodules had significantly lower ADC_SD_ (*P* = 0.005), ADC_SIR_ (*P* = 0.008), ADC_min_ (*P* < 0.001), and ADC_mean_ (*P* < 0.001) than benign nodules. The ICCs of DWI_SD_, DWI_SIR_, ADC_SD_, ADC_SIR_, ADC_min_ and ADC_mean_ were 0.776, 0.758, 0.720, 0.923, 0.789, 0.783 and 0.743, respectively. ADC_min_ was the best-performing parameter with an AUC of 0.933 (0.874—0.992). Figure 3a and Table 3 represent the ROC curves and diagnostic performance metrics at the optimal threshold of relevant individual parameters, respectively.

**Table 2:** Comparisons of quantitative DWI parameters to identify malignant thyroid nodules

Variables	Benign (*n* = 62)	Malignant (*n* = 20)	Univariate analysis		Multivariate analysis		ICC
			OR (95%CI)	*P* value	OR (95%CI)	*P* value	
DWI_SD_	18.64 ± 10.32	36.23 ± 26.02	1.070(1.026–1.117)	0.002*			0.776
DWI_SIR_	1.24 ± 0.54	1.70 ± 0.68	3.427(1.395–8.416)	0.007*	4.526(1.084–18.892)	0.038*	0.758
ADC_SD_	124.66 ± 53.37	83.63 ± 42.71	0.979(0.964–0.994)	0.005*			0.720
ADC_SIR_	1.35 ± 0.28	1.14 ± 0.31	0.061(0.008–0.489)	0.008*			0.923
ADC_min_ (× 10^−3^mm^2^/s)	1.42 ± 0.23	0.88 ± 0.30	< 0.001 (< 0.001–0.007)	< 0.001*	< 0.001 (< 0.001–0.007)	< 0.001*	0.789
ADC_mean_ (× 10^−3^mm^2^/s)	1.96 ± 0.36	1.36 ± 0.43	0.015(0.002–0.115)	< 0.001*			0.783
ADC_VSD_	121.75 ± 65.97	98.26 ± 45.86	0.992(0.982–1.003)	0.143			0.743

*Abbreviations*: *DWI* Diffusion weighted imaging, *ADC* Apparent diffusion coefficient, *SD* Standard deviation, *OR* OddsRatio, *CI* Confidence interval, *SIR* Signal intensity rate, *ADC*_*min*_ Minimum apparent diffusion coefficient value, *ADC*_*mean*_ Mean apparent diffusion coefficient value, *ADC*_*VSD*_ Standard deviation of apparent diffusion coefficient value, *ICC* Intraclass correlation coefficient

* *P* < 0.05

**Table 3:** The diagnostic performance of meaningful single DWI parameters to identify malignant thyroid nodules

Variables	Threshold value	Yoden’s index	Accuracy%	Sensitivity%	Specificity%	PPV%	NPV%	AUC
DWI_SD_	> 19.10	0.477	70.7	80.0	67.7	44.4	91.3	0.751(0.604–0.898)
DWI_SIR_	> 1.20	0.432	62.2	90.0	53.2	38.3	94.3	0.734(0.604–0.864)
ADC_SD_	< 88.35	0.476	75.6	65.0	77.4	48.1	87.3	0.762(0.637–0.887)
ADC_SIR_	< 1.25	0.397	63.4	75.0	59.7	37.5	88.1	0.710(0.574–0.847)
ADC_min_ (× 10^–3^ mm^2^/s)	< 1.13	0.735	90.2	80.0	93.5	80.0	93.5	0.933(0.874–0.992)
ADC_mean_ (× 10^–3^ mm^2^/s)	< 1.47	0.535	85.3	60.0	93.5	75.0	87.9	0.851(0.759–0.944)

*Abbreviations*: *DWI* Diffusion weighted imaging, *ADC* Apparent diffusion coefficient, *PPV* Positive predictive value, *NPV* Negative predictive value, *AUC* Area under the receiver operating characteristic curve, *SD* Standard deviation, *SIR* Signal intensity rate, *ADC*_*min*_ Minimum apparent diffusion coefficient value, *ADC*_*mean*_ Mean apparent diffusion coefficient value, *ADC*_*VSD*_ Standard deviation of apparent diffusion coefficient value, *ACR-TIRADS* American College of Radiology Thyroid Imaging Reporting and Data System

**Fig. 4:**
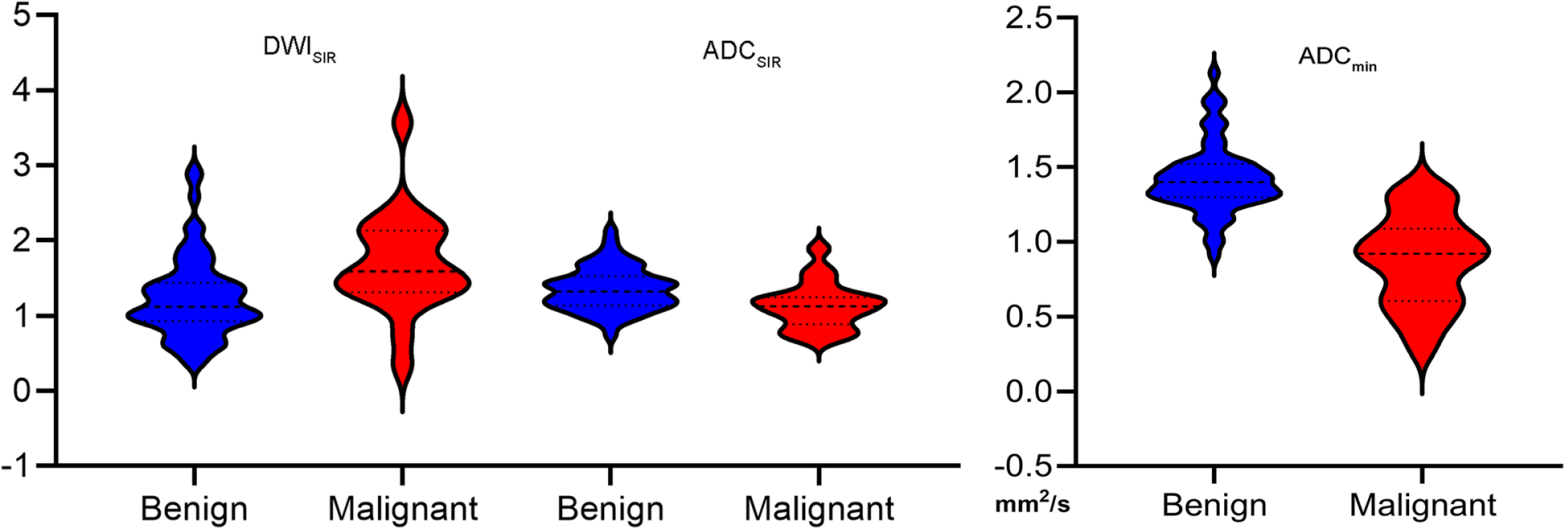
The Violin chart of DWI_SIR_, ADC_SIR_ and ADC_min_. Abbreviations: DWI, diffusion weighted imaging; ADC, apparent diffusion coefficient; SIR, signal intensity rate; ADC_min_, minimum apparent diffusion coefficient value

The optimal threshold values were 1.13 × 10^–3^ mm^2^/s for ADC_min_, 1.25 for ADC_SIR_, and 1.20 for DWI_SIR_, showing their distribution in Fig. 4. For benign and malignant nodules, DWI_SIR_, ADC_SIR_, and ADC_min_ overlapped; however, malignant nodule ADC_min_ was comparatively low.

### Comparison of the diagnostic performance with ACR-TIRADS

**Fig. 5:**
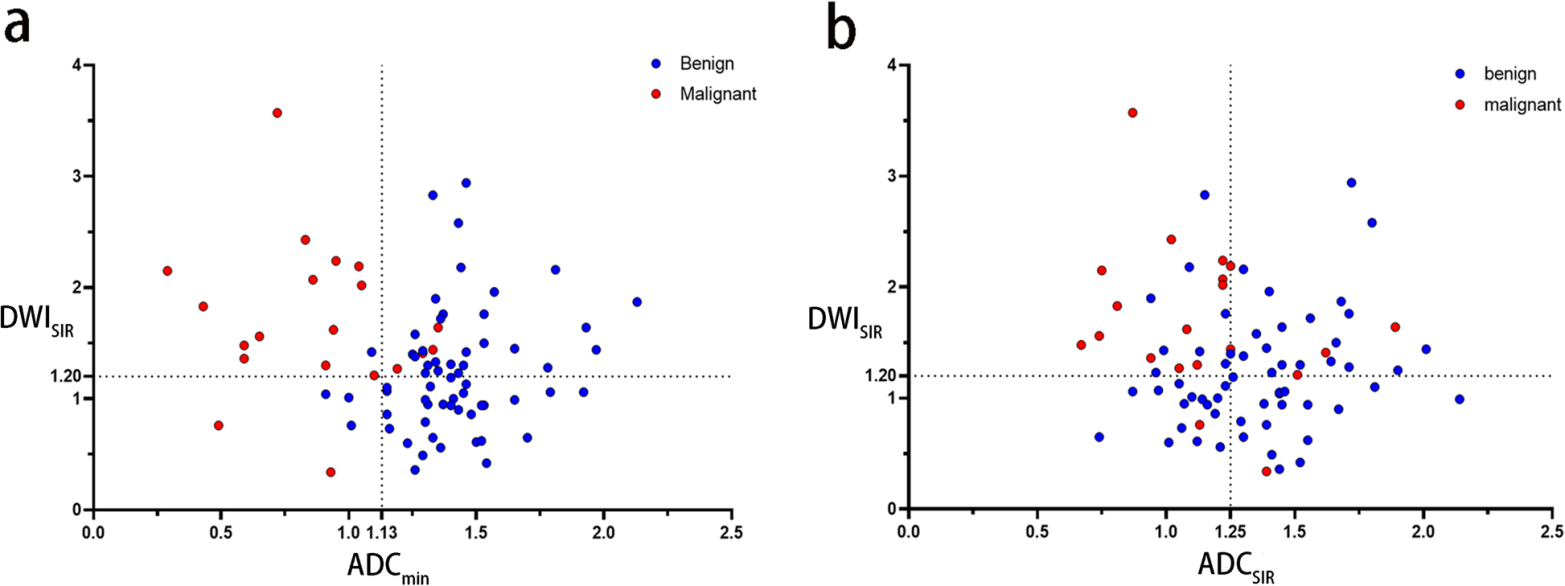
The grouped scatter plots of the two combined thresholds models. The blue dots are benign lesions, and the red dots are malignant lesions. **a** is the scatter plot of DWI_SIR_ and ADC_min_ of the lesions. **b** is the scatter plot of DWI_SIR_ and ADC_min_ of the lesions. Abbreviations: DWI, diffusion weighted imaging; ADC, apparent diffusion coefficient; SIR, signal intensity rate; ADC_min_, minimum apparent diffusion coefficient value

Table 4 and Fig. 3b depict the diagnostic performance of the multivariate prediction model, combined threshold model, and ACR-TIRADS for malignant and benign thyroid nodules. The multivariate prediction model had the best diagnostic performance with an AUC of 0.946 (0.896–0.996) at a cutoff value of 0.198, which was higher than the AUC achieved by the combined threshold model (DWI_SIR_ and ADC_min_), with insignificance difference (*P* = 0.500). The AUC of combined threshold model (DWI_SIR_ and ADC _SIR_) and ACR-TIRADS were 0.777 (0.648–0.907) and 0.722 (0.588–0.857), respectively. Figure 5 reveals the grouped scatter plots of the two combined threshold models, and Table S3 summarizes the Delong test results for AUC comparison among different models.

**Table 4:** The diagnostic performance of models based on quantitative DWI parameters to identify malignant thyroid nodules

Approaches		Accuracy%	Sensitivity%	Specificity%	PPV%	NPV%	AUC	Unnecessary biopsy rate %
Prediction model (cutoff value = 0.198)		87.8	85.0	88.7	70.8	94.8	0.946 (0.896–0.996)	29.2
Combined thresholds	DWI_SIR_ (threshold value = 1.20)	93.9	75.0	100.0	100.0	92.5	0.875 (0.759–0.991)	0.0
	ADC_min_ (threshold value = 1.13 × 10^-3^ mm^2^/s)							
	DWI_SIR_ (threshold value = 1.20)	81.7	70.0	85.5	60.9	89.8	0.777 (0.648–0.907)	39.1
	ADC_SIR_ (threshold value = 1.25)							
ACR-TIRADS (cutoff: TR ≥ 4)		77.8	66.7	77.8	43.8	90.0	0.722 (0.588–0.857)	56.3

*Abbreviations: DWI* Diffusion weighted imaging, *ADC* Apparent diffusion coefficient, *PPV* Positive predictive value, *NPV* Negative predictive value, *AUC* Area under the receiver operating characteristic curve, *SD* Standard deviation, *SIR* Signal intensity rate, *ACR-TIRADS* American College of Radiology Thyroid Imaging Reporting and Data System, *TR* TI-RADS

The sensitivity (90.0%) and NPV (96.6%) were the highest in the multivariate prediction model. The results showed three false negative lesions, all follicular thyroid carcinoma, and seven false positive lesions: three nodular goiters, three adenomatous nodular goiters, and one adenoma. The best specificity and PPV (both 100%) were achieved by the combined threshold model (DWI_SIR_ and ADC_min_), where five false negative lesions were all follicular thyroid carcinomas. The combined DWI_SIR_ and ADC_min_ had the lowest unnecessary biopsy rate with no false positive cases. The accuracy, sensitivity, specificity, PPV and NPV of the combined thresholds model (DWI_SIR_ and ADC_SIR_) were 81.7%, 70%, 85.5%, 60.9% and 89.2%, respectively. Compared with ACR-TIRADS, the quantitative DWI parameter-based models significantly improved differentiating benign and malignant thyroid nodules.

## Discussion

This study proposed diagnostic models based on quantitative DWI parameters without enhancement to differentiate between benign and malignant thyroid nodules larger than 4 cm. The combined threshold model (DWISIR and ADCmin) demonstrated satisfactory diagnostic efficacy with significantly reduced unnecessary biopsy rate.

ADC map, derived from DWI, measures water diffusion in tissue and provides a new imaging biomarker for the diagnosis of benign and malignant tumours [25]; it has proven to be effective in predicting tumor malignancy [26]. Malignant thyroid nodules had much lower ADC values than the benign thyroid nodules, according to numerous studies [22, 27, 28], but they were for nodules smaller than 4 cm. We measured ADC_min_, ADC_mean_ and ADC_SD_, and found that the former two were associated with malignancy. In a meta-analysis of 2137 thyroid nodules, ADC_mean_ was demonstrated to be a useful tool for differentiating between benign and malignant thyroid tumors and should be used in routine preoperative clinical testing. The ADC_mean_ was 1.88 × 10^–3^ mm^2^/s in the benign nodules and 1.15 × 10^–3^ mm^2^/s in the malignant [22]; herein, for lesions larger than 4 cm, the ADC_mean_ value was 1.74 × 10^–3^ mm^2^/s in benign nodules and 1.08 × 10^–3^ mm^2^/s in malignant nodules. ADC_min_ was found to be an independent predictor in our study. One study of benign and malignant lymph node metastases in the breast indicated that ADC_mean_ had better diagnostic efficacy than ADC_min_ [29]. However, in a study of benign and malignant prostate tumors, ADC_min_ was found to be superior to ADC_mean_ [30], which was similar to our results. Due to the multicollinearity between ADC_min_ and ADC_mean_ in the combined threshold model, we chose ADC_min_ as it had a greater AUC value in the univariate analysis.

We also investigated the signal intensity-related parameters of DWI and corresponding ADC images on PACS. Leila et al. [31] reported that static MRI measurements like signal intensity and heterogeneity were unuseful in distinguishing between benign and malignant lesions. However, Wang et al. [32] showed that DWI_SIR_ was lower in benign nodules than in malignant ones, corroborating our findings. Our study found that DWI_SIR_ was another independent predictor of malignant nodules, with malignant nodules showing a higher DWI_SIR_. which corroborated with our findings. DWI_SIR_ can complement ADC_min_, and using the combined threshold model (DWI_SD_ and ADC_min_) reduced the number of false positive cases by four and resulted in the highest specificity (100%). According to ACR-TIRADS, the biopsy is recommended for lesions with TR ≥ 4 and diameter > 1.5 cm. Therefore, all nodules with TR ≥ 4 in this study required biopsy to determine their benignity and malignancy, and the unnecessary biopsy rate was up to 56.3%. The combined threshold model (DWI_SIR_ and ADC_min_) had the lowest unnecessary biopsy rate. DWI may be performed before preparation for biopsy and assisted in determining the need for biopsy and surgery based on quantitative DWI. Quantitative DWI may become a method of thyroid nodules larger than 4 cm surveillance to aid clinicians in their medical decisions.

In addition to ADC_min_, we also measured ADC_SIR_, which is easily available on PACS. ADC_SIR_ was lower in malignant nodules than in benign nodules significantly, which may be because malignant lesions have dense parenchymal cells, narrow cell spaces, and relatively limited extracellular water molecule diffusion. While the combined threshold model (DWI_SIR_ and ADC_SIR_), while it was inferior to the combined threshold model (DWI_SIR_ and ADC_min_), it still resulted in nine fewer false positive cases than ACR-TIRADS alone.

This study found that individual parameters performed poorly in distinguishing the benignity of follicular thyroid neoplasm. The difference between follicular carcinoma and benign follicular neoplasm is the invasion of the envelope invasion, which can only be detected by postoperative pathology. Our measurements are limited to the substantial lesion component, and this pathological level of distinction is difficult to detect on images.

Although this study provided important insights, there were several limitations. First, selection bias is unavoidable in a retrospective observational study. Second, the sample size was relatively small, necessitating additional validation. Third, reviewing static ultrasound images and reports may differ from real-life clinical practice, introducing some bias. Finally, the study only used a b-value of 800 s/mm2. Therefore, more research must be conducted with various b-values to find the best value.

In conclusion, quantitative DWI parameters can separate benign from malignant thyroid nodules larger than 4 cm. The multivariate prediction and combined threshold model (DWI_SIR_ and ADC_min_) demonstrated satisfactory diagnostic performance. Our results suggested that quantitative DWI parameters can assess benign and malignant nodules with sizes larger than 4 cm and assist clinicians in pre-operative decision-making.

### Supplementary Information

**Additional file 1: Table S1. **Parameters of MRI Sequence. **Figure S1.** Flowchart of combined thresholds models. **Table S2. **Pathological types. **Table S3.** Delong test results for AUCs between different models.

## Data Availability

The data sets generated and/or analyzed in the current study were not made public because patients' personal information was included. Available from the corresponding author upon reasonable request.

## Abbreviations

DWI: Diffusion-weighted imaging
ADC: Apparent diffusion coefficient
ROC: Receiver operating characteristic
AUC: Area under the receiver operating characteristic curve
PPV: Positive predictive value
NPV: Negative predictive value
ACR-TIRADS: American College of Radiology Thyroid Imaging Reporting and Data System
DWI_SIR_: Diffusion-weighted imaging signal intensity rate
ADC_min_: Minimum apparent diffusion coefficient value
TNM: Tumor, lymph node, metastasis
AJCC: American Joint Committee on Cancer
MRI: Magnetic resonance imaging
T1WI: T1-weighted imaging
T2WI: T2-weighted imaging
SS-SE-EPI: Single-shot spin-echo echo-planar imaging
PACS: Picture archiving and communication system
ROI: Region of interest
DWI_SI_: Diffusion-weighted imaging signal intensity
ADC_SI_: Apparent diffusion coefficient signal intensity
ADC_mean_: Mean apparent diffusion coefficient value
ADC_min_: Minimum apparent diffusion coefficient value
DWI_SD_: Signal intensity standard deviation
ADC_SD_: Signal intensity standard deviation
ADC_VSD_: Standard deviation of apparent diffusion coefficient value
DWI_SIR_: Diffusion-weighted imaging signal intensity rate
ADC_SIR_: Apparent diffusion coefficient signal intensity rate
ICC: Intraclass correlation coefficient
PTC: Papillary thyroid carcinoma
FTC: Follicular thyroid carcinoma
