# Supplementary material for: Quantitative evaluation of diffusion-weighted MRI for differentiating benign and malignant thyroid nodules larger than 4 cm
Source: BMC Med Imaging. 2023 Dec 13;23:212. doi: 10.1186/s12880-023-01141-z (PMC10720093; doi:10.1186/s12880-023-01141-z)
Supplement: Supplementary file 1 — Additional file 1: Table S1. Parameters of MRI Sequence. Figure S1. Flowchart of combined thresholds models. Table S2. Pathological types. Table S3. Delong test results for AUCs between different models. [file 12880_2023_1141_MOESM1_ESM.docx]

**Supplementary Materials**

**Table S1** Parameters of MRI Sequence

| Sequence | Plane | TR | TE | Slice thickness | Gap between slices | NEX | FOV | Matrix size | Sequence |
| --- | --- | --- | --- | --- | --- | --- | --- | --- | --- |
| T2WI with fat suppression | coronal | 1280 | 85 | 3-4 | 1 | 4 | 18 | 288×192 | FRFSE |
| T1WI | axial | 460 | 8 | 3-4 | 0.5 | 2 | 25 | 288×192 | FSE |
| T2WI with fat suppression | axial | 3000 | 85 | 3-4 | 0.5 | 4 | 25 | 320×224 | FRFSE |
| DWI | axial | 6550 | minimum | 3-4 | 0.5 | 6 | 25 | 128×128 | SS-EPI |
| Contrast-enhanced T1WI | axial | 5.7 | 1.7 | 3-4 | 0.5 | 1 | 25 | 192×256 | FSPGR |
| Units |  | ms | ms | mm | mm |  | cm |  |  |

**Abbreviations:** TR, repetition time; TE, echo time; NEX, number of excitations; FOV, field of view; T2WI, T2WI-weighted image; T1WI, T1-weighted image; DWI, diffusion-weighted imaging; FRFSE, fast recovery fast spin echo; FSE, fast spin echo; SS-EPI, single shot echo-planar imaging; FSPGR, fast spoiled gradient echo.

**Combined thresholds models**

The parameters of ADC_min_ and DWI_SIR_ were combined to provide a single integrated diagnostic marker. Thresholds were established by receiver operating characteristic analysis (ROC) curves to maximize the sensitivity and specificity of benign and malignant lesions. The threshold for ADC_min_ is 1.13×10^-3^ mm^2^/sec and for DWI_SIR_ is 1.20. If both ADC_min_ was less than 1.13×10^-3^ mm^2^/sec and DWI_SIR_ was greater than 1.20, the combination thresholds model is determined to be malignant; otherwise, it is determined to be benign. Similarly, combined DWI_SIR_ with ADC_SIR_ to provide a single integrated diagnostic marker. The threshold for ADC_SIR_ is 1.25. If both ADC_SIR_ was less than 1.25 and DWI_SIR_ was greater than 1.20, the combination thresholds model is determined to be malignant; otherwise, it is determined to be benign. The diagnostic efficacy of the combined thresholds models was then calculated and compared with ACR-TIRADS.


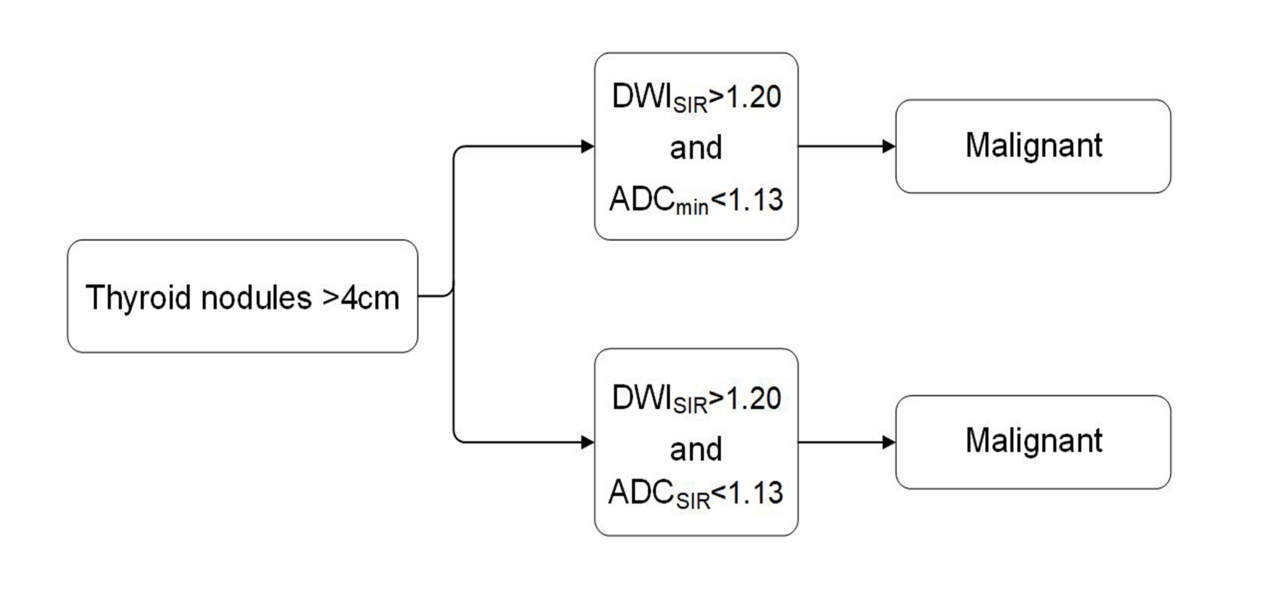


**Figure S1**. Flowchart of combined thresholds models

**Abbreviations:** DWI, diffusion weighted imaging; ADC, apparent diffusion coefficient; SIR, signal intensity rate; ADC_min_, minimum apparent diffusion coefficient value.

**Table S2** Pathological types

| Pathological pattern | N |
| --- | --- |
| Papillary thyroid carcinoma | 5 (6.1) |
| Follicular thyroid carcinoma | 13 (15.9) |
| Undifferentiated carcinoma | 2 (2.4) |
| Nodular goiter | 25 (30.5) |
| Adenoma | 17 (20.7） |
| Adenomatous nodular goiter | 20 (24.4) |

The data are presented as number of patients with the percentage in parentheses.

**Abbreviations:** N, number.

**Table S3** Delong test results for AUCs between different models

| Model | P |
| --- | --- |
| Prediction model **VS** Combined thresholds model (DWI_SIR_ and ADC_SIR_) | 0.036* |
| Prediction model **VS** Combined thresholds model (DWI_SIR_ and ADC_min_) | 0.500 |
| Combined thresholds model (DWI_SIR_ and ADC_SIR_)  **VS** Combined thresholds model (DWI_SIR_ and ADC_min_) | 0.018* |
| ACR TIRADS **VS** Prediction model | 0.050* |
| ACR TIRADS **VS** Combined thresholds model (DWI_SIR_ and ADC_SIR_) | 0.475 |
| ACR TIRADS **VS** Combined thresholds model (DWI_SIR_ and ADC_min_) | 0.041* |

**Abbreviations:** DWI, diffusion weighted imaging; ADC, apparent diffusion coefficient; AUC, area under the receiver operating characteristic curve; SIR, signal intensity rate; ACR-TIRADS, American College of Radiology Thyroid Imaging Reporting and Data System.
